# More Than Meets the Eye: A Rare Case of Posterior Scleritis Masquerading as Orbital Cellulitis

**DOI:** 10.7759/cureus.8177

**Published:** 2020-05-18

**Authors:** Christopher Hogan, George Vakros, Rebecca Jones, Sanjana Bhalla, Katherine McVeigh

**Affiliations:** 1 Otolaryngology, West Middlesex University Hospital, London, GBR; 2 Vitreoretinal Surgery/Comprehensive Ophthalmology, Moorfields Eye Hospital, London, GBR; 3 Ophthalmology, Cheltenham General Hospital, London, GBR; 4 Otolaryngology, St Mary's Hospital, London, GBR; 5 Ophthalmology, Bristol Eye Hospital, Bristol, GBR

**Keywords:** posterior scleritis, periorbital cellulitis

## Abstract

Posterior scleritis is a rare sight-threatening condition that typically presents with an acutely painful eye, often with associated reduced visual acuity. Diagnosis can be challenging and requires specialist ophthalmological assessment. Consequences of delayed treatment include permanent loss of vision. We present a case of posterior scleritis initially managed as periorbital cellulitis. We highlight the importance of broad differential diagnoses when assessing painful periorbital swelling, and present a review of current management strategies for posterior scleritis.

## Introduction

Scleritis is a painful, sight-threatening, inflammatory condition of the sclera. It typically presents with an acutely red and painful eye and can be associated with decreased visual acuity. Inflammation of the periorbital soft tissues and ophthalmoplegia are not typically associated with posterior scleritis [1]. We present a case of posterior scleritis masquerading as periorbital cellulitis, with associated periorbital oedema and ophthalmoplegia. We are aware of only two other such cases in the literature.

This case report serves to educate emergency physicians, otolaryngologists, paediatricians and ophthalmologists of this rare differential diagnosis. We believe that non-response to intravenous antibiotics, combined with the absence of CT evidence of sinusitis, should lead healthcare practitioners to have a high index of suspicion for alternative diagnoses. As a number of these differential diagnoses are associated with significant morbidity, and rarely mortality, we highlight that early involvement of ophthalmologists is a vital aspect of the management of acute painful periorbital swelling.

## Case presentation

A 59-year-old woman presented to the emergency department (ED) with a seven-day history of a painful right eye, associated with periorbital swelling and an acute deterioration in vision. There was no history of injury, sinusitis or stye. She did not complain of sinonasal symptoms.

**Figure 1:**
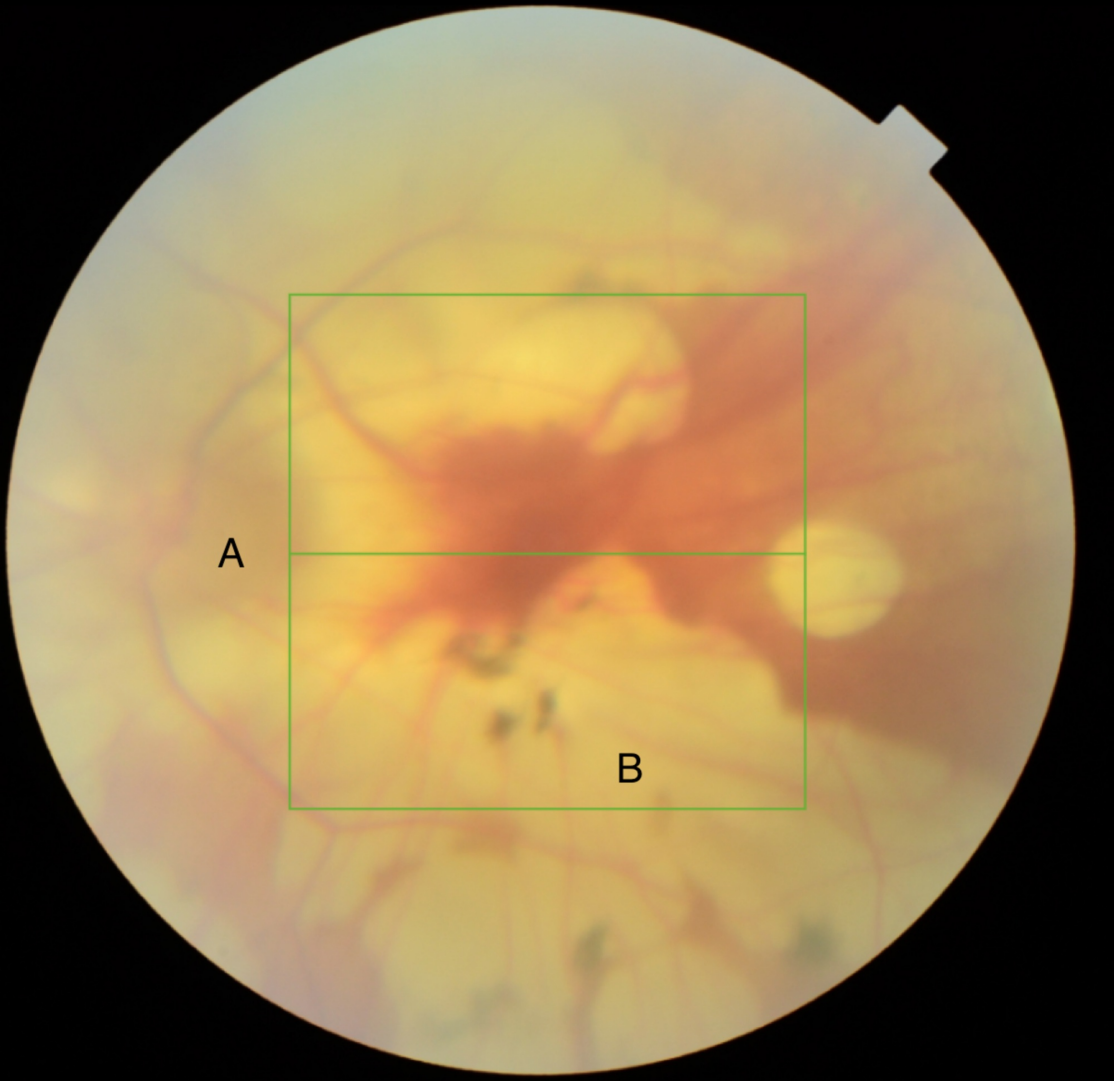
An optical coherence tomography image of this patient's left retina, demonstrating features consistent with myopia: a tilted optic disc (A) and retinal atrophy (B).

Her ophthalmological history included myopia and a previous right retinal detachment, repaired surgically with scleral buckling. On examination, her visual acuity was reduced (6/48) in the affected eye. She had conjunctival chemosis, proptosis and periorbital oedema of the right eye. The sclera was white. She had restricted abduction of the eye, and fundoscopy showed a tilted optic disc with retinal atrophy, changes associated with high myopia (Figure 1). Systemic examination was normal, and she was apyrexial.

**Figure 2:**
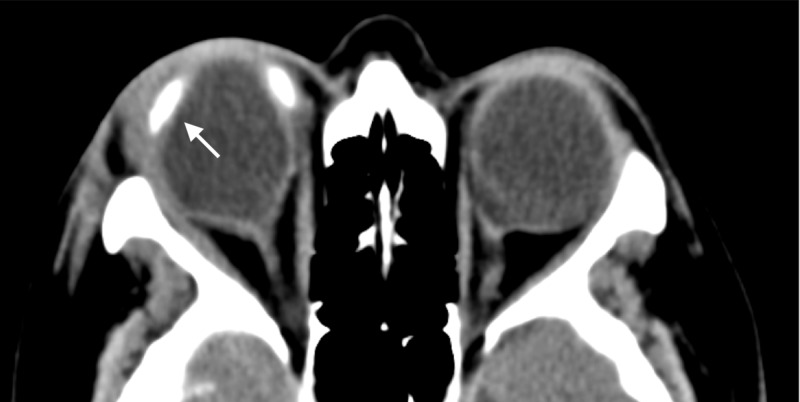
An axial CT image of the orbits. The radiopaque scleral buckle (white arrow) can clearly be seen encircling the right globe. There is no radiological evidence of any intra-orbital pathology; specifically no abscess or mass lesion. The corresponding ethmoid sinus is noted to be air-filled, without opacification.

**Figure 3:**
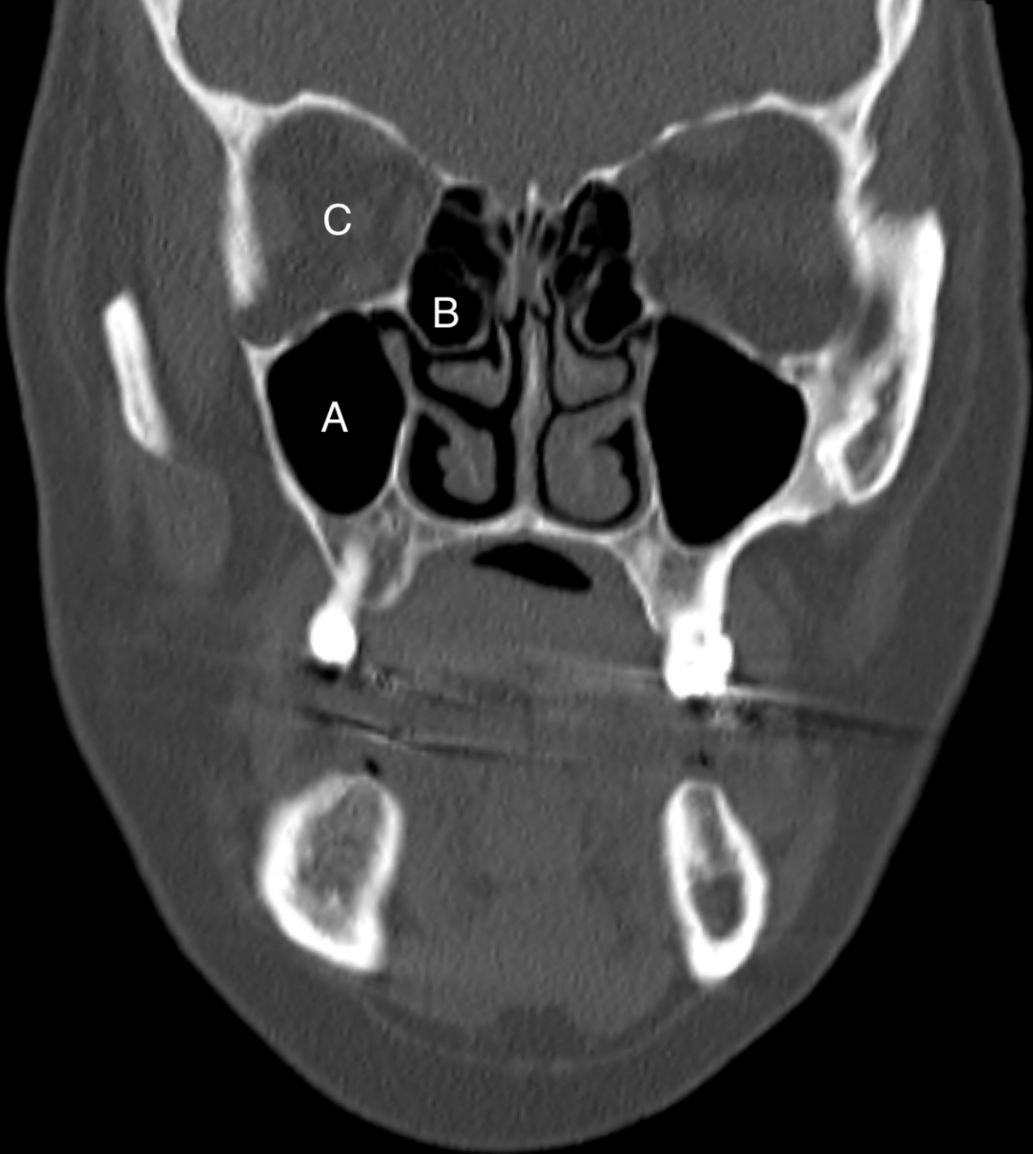
A coronal CT image of the paranasal sinuses, orbits and brain of this patient. There are no signs of sinusitis: no mucosal thickening or opacification in any of the visible sinuses, including the right maxillary sinus (A) and the right ethmoid sinus (B). The orbit (C) does not demonstrate any intra-orbital pathology.

Investigations revealed a raised C-reactive protein (72 mg/L), raised erythrocyte sedimentation rate (64 mm/h), a normal white cell count and mild thrombocythemia (426 x 10^9^/L). Thyroid, liver and renal function tests were normal. CT of the paranasal sinuses and orbits excluded any orbital pathology (Figure 2) or sinusitis (Figure 3). She was admitted and initiated on intravenous broad-spectrum antibiotics, empirical treatment for periorbital cellulitis.

During admission, her symptoms deteriorated despite antibiotic therapy. Her visual acuity decreased to counting fingers on the right, and the periorbital oedema, chemosis and proptosis did not improve. The ophthalmology team was consulted, who identified macular oedema and revised the diagnosis to one of posterior scleritis.

The patient was commenced on flurbiprofen 100 mg TDS along with topical steroids and mydriatics. Within three days, the oedema improved and the conjunctival chemosis had almost settled. At a follow-up clinic appointment one week after discharge, she was asymptomatic, with her vision at baseline. On examination, the periorbital oedema had resolved and there was no ophthalmoplegia.

Due to the severity of her presentation, she was referred to a tertiary centre for further investigation. A full autoimmune blood screen was performed and was negative. No underlying systemic inflammatory disease was identified, leaving the most likely underlying aetiology for her scleritis being her previous scleral buckle surgery. She was commenced on methotrexate, and six months after her initial presentation she remained asymptomatic. The pathophysiology of ophthalmoplegia was thought to be localized inflammation of the extraocular muscles secondary to the posterior scleral inflammation, and did not recur. 

## Discussion

Differential diagnoses

The presentation of scleritis can vary significantly depending on the anatomical site involved (anterior and/or posterior sclera), and the extent of scleral inflammation. The pain associated with scleritis is exacerbated with movements of the eye, as extraocular muscles insert into the sclera. Patients often also describe disturbed sleep patterns, a hallmark of the disease, as rapid eye movement (REM) sleep can lead to exacerbation of symptoms. The scleral inflammation can be described, in increasing severity, from diffuse to nodular to necrotizing [1].

Periorbital cellulitis is an infection of the skin and soft tissues of the eye. Periorbital cellulitis can be associated with life- and sight-threatening complications, graded by the Chandler classification: orbital cellulitis, periosteal abscess, orbital abscess and cavernous sinus thrombosis [2]. Periorbital cellulitis typically presents as an acutely swollen painful eye. Signs of orbital complications can include decreased visual acuity, proptosis, relative afferent pupillary defect, ophthalmoplegia and pyrexia. Periorbital cellulitis is typically a complication of bacterial ethmoid sinusitis and is found more commonly in children. There are therefore usually preceding sinonasal symptoms such as nasal obstruction, rhinorrhoea, midfacial pain and hyposmia. The most common causative pathogens in the UK are *Staphylococcus aureus* and *Streptococcus *spp.

The diagnosis of painful periorbital swelling also includes thyroid eye disease and orbital masses. Symptoms of thyroid eye disease include proptosis, pain, excessive tearing, blurring of vision and diplopia. Rarely, if it remains untreated, compression of the optic nerve secondary to the orbital inflammation can lead to loss of vision. In addition, other symptoms of hyperthyroidism are usually present. Graves’ ophthalmopathy occurs in 20%-25% of patients with Graves’ disease [3]. An orbital mass can also present with acute painful periorbital swelling, but this is rare. Orbital masses can include neuroblastoma, rhabdomyosarcoma, haemangioma and leukaemia. Symptoms tend to be slowly progressive than those of the other differential diagnoses, and are often associated with malignancy-related constitutional symptoms.

Aetiopathogenesis of scleritis

Scleritis is an immune-mediated condition, and 50% of patients with scleritis have an associated systemic inflammatory condition, either infectious or immune in nature [4]. The pathophysiology of scleritis is not well defined; however, studies have suggested immune complex deposition and neutrophil invasion of vessel walls leading to fibrinoid necrosis and thrombosis of blood vessels inducing inflammation of the sclera.

The most common immune-related condition is rheumatoid arthritis (RA). In RA, the aetiology of the scleritis is a rheumatoid vasculitis, which is a complication of RA characterized by small and medium vessel vasculitis [5]. In such patients, rheumatoid vasculitis leads to necrosis, occlusion of blood vessels and tissue ischaemia in a manner similar to systemic vasculitides. The scleritis in such cases is necrotizing in nature. The most common non-rheumatoid vasculitides associated with scleritis are antineutrophil cytoplasmic antibody (ANCA)-positive vasculitides, such as granulomatosis with polyangiitis (previously known as Wegener’s granulomatosis), microscopic polyangiitis and Churg-Strauss syndrome. Watkins et al. identified scleritis as the most common ocular symptom of ANCA-positive vasculitides, followed by uveitis [6]. Connective tissue diseases related to systemic lupus erythematosus (SLE) are also linked with scleritis. The strongest ocular association of SLE, however, is not scleritis but keratoconjunctivitis sicca [7].

The most common infectious associations of scleritis are varicella zoster virus and herpes zoster virus. Several case reports have also highlighted syphilis as the cause of scleritis, mostly in immunosuppressed patients [8]. The condition occurs in acquired syphilis and mainly during the secondary and tertiary stages. Other infective agents of note include fungal pathogens, *Staphylococcus *spp*.* and *Pseudomonas aeruginosa *[9].

Orbital trauma or surgery is another documented association of scleritis. Studies have reported cataract surgery, scleral buckling for retinal detachment, strabismus correction surgery and pterygium excision as surgical procedures that can be associated with scleritis [10].

Investigation

Scleritis should be suspected in every acutely painful red eye, especially if tests for alternative diagnoses are equivocal or if the pain is especially severe at night. Once a diagnosis of scleritis is clinically suspected, a slit lamp examination can identify inflammation of the anterior sclera: conjunctival chemosis, vessel dilation and tortuosity. B-scan orbital ultrasonography can elucidate the extent of the inflammation and the presence or absence of posterior scleral involvement, defined as involvement of sclera posterior to the insertion of the rectus muscles. 

Although 50% of scleritis cases are idiopathic, certain investigations can help diagnose underlying causes, if present. Patients with systemic inflammatory conditions frequently have abnormal white cell counts, platelet counts or haematocrit levels. Routine blood tests such as liver function tests (LFTs) and urea and electrolytes (U&Es) may reveal associated visceral damage due to underlying associated autoimmune disease processes, such as lupus hepatitis or glomerulonephritis [11]. For the latter, urinalysis with microscopy is essential as glomerulonephritis is a common occurrence in granulomatosis with polyangiitis and SLE. Acute-phase proteins, such as erythrocyte sedimentation rate and C-reactive protein, tend to be high in systemic inflammatory conditions and should be requested. These markers can also be used in monitoring disease activity and response to therapy [12].

In addition to the initial baseline tests, specific serological tests for underlying autoimmune conditions are indicated. Though not specific, a high serum level of rheumatoid factor (RF) is indicative of rheumatoid vasculitis [13]. Raised levels of RF are also associated with various other autoimmune conditions, such as RA, mixed connective tissue disease and SLE, all associated with scleritis. Anti-cyclic citrullinated peptides (anti-CCP antibodies) can also be used to help diagnose RA [14].

ANCAs are associated with various subtypes of granulomatosis with polyangiitis; however, the absence of ANCAs does not exclude vasculitis as they can be absent in 40% of cases [15]. Antinuclear antibody (ANA) testing is also useful for the evaluation of connective tissue diseases related to SLE.

Chest radiographs can help identify inflammatory nodules or infiltrates that can be associated with vasculitis. CT and MRI of the orbits can be particularly useful in identifying orbital masses or pseudotumours, occasionally seen in granulomatosis with polyangiitis [16].

Management

The management of scleritis is dependent on many factors, but a multidisciplinary approach involving ophthalmology and rheumatology is often required for optimal management. Complications of scleritis include corneal ulcers, glaucoma, cataract formation, retinal detachment and globe rupture [1]. Each of these is potentially sight-threatening, underlining the need for expert management.

Oral non-steroidal anti-inflammatory drugs (NSAIDs) with topical steroids and mydriatics are the first line of treatment. In cases where these measures do not achieve the desired response, systemic immunosuppression in the form of intravenous corticosteroids, or other immunomodulatory agents such as methotrexate, can improve disease relapses and control the underlying aetiology. It is also important to consider the presence or absence of associated systemic inflammatory processes in the treatment plan, and hence the importance of rheumatological input for these patients.

Generally, diffuse and nodular subtypes of scleritis respond well to NSAIDs (indomethacin and flurbiprofen are both frequently used); however, due to treatment failure or occasional unacceptable adverse effects, corticosteroids can be trialled. In comparison, more than 60% of patients with posterior or necrotizing scleritis require either glucocorticoids or other immunosuppressive agents to control their disease [17].

Where corticosteroids have no significant effect, further immunosuppressive medications can be trialled. Rituximab and cyclophosphamide are generally considered first-line agents due to their relative successes in the management of systemic inflammatory conditions, such as granulomatosis with polyangiitis [18]. Cyclosporine, mycophenolate mofetil and methotrexate are also available as steroid-sparing agents. When scleritis is found to have a directly infective aetiology, the addition of the pathogen-specific treatment agent is an important consideration [19].

The lack of evidence on the efficacy of immunosuppressive agents for scleritis emphasizes the importance of expert management of severe or resistant disease. Newer agents such as tocilizumab and adalimumab have been shown, on a case-report basis, to induce complete remission in treatment-resistant scleritis, highlighting the need for further research into the management of this disease [20].

## Conclusions

This patient’s initial presentation was suggestive of orbital cellulitis, which physicians should have a high index of suspicion for, due to its significant associated morbidity and mortality. However, the patient’s symptoms and signs did not improve with intravenous antibiotics, and microbiological investigations did not reveal an infective agent. Furthermore, a CT scan of the orbits did not demonstrate any evidence of sinonasal or orbital disease. In addition to the exclusion of orbital cellulitis, the CT scan excluded the possibility of an extra-orbital infiltrative lesion. The speed of onset and nature of this patient’s symptoms do not correlate with Grave’s ophthalmopathy, which was further excluded with thyroid function testing.

Aside from the exclusion of these important differential diagnoses, there were features that assist with the diagnosis of scleritis. The history of scleral buckling, a known risk factor for scleritis, should lead physicians to include scleritis in their differential diagnoses. The rapid clinical response to oral NSAIDs and mydriatics, to complete resolution of symptoms, confirmed posterior scleritis as the diagnosis. This case report highlights the importance of the consideration of the wide range of differential diagnoses for acute painful periorbital swelling. 
